# Exploring the Characteristic Aroma of Beef from Japanese Black Cattle (Japanese Wagyu) via Sensory Evaluation and Gas Chromatography-Olfactometry

**DOI:** 10.3390/metabo11010056

**Published:** 2021-01-15

**Authors:** Shuji Ueda, Minoru Yamanoue, Yasuhito Sirai, Eiji Iwamoto

**Affiliations:** 1 Department of Agrobioscience, Graduate School of Agricultural Science, Kobe University, Kobe, Hyogo 657-8501, Japan; yamanoue@kobe-u.ac.jp (M.Y.); shirai@kobe-u.ac.jp (Y.S.); 2 Hokubu Agricultural Technology Institute Hyogo Prefectural Technology Center for Agriculture, Forestry and Fisheries, Asago, Hyogo 669-5254, Japan; eiji_iwamoto@pref.hyogo.lg.jp

**Keywords:** Japanese Black cattle, metabolomics, GC–olfactometry, Wagyu beef aroma

## Abstract

Beef from Japanese Black cattle (Japanese Wagyu) is renowned for its flavor characteristics. To clarify the key metabolites contributing to this rich and sweet aroma of beef, an omics analysis combined with GC-olfactometry (GC-O) and metabolomics analysis with gas chromatography–mass spectrometry (GC-MS) were applied. GC-O analysis identified 39 odor-active odorants from the volatile fraction of boiled beef distilled by solvent-assisted flavor evaporation. Eight odorants predicted to contribute to Wagyu beef aroma were compared between Japanese Black cattle and Holstein cattle using a stable isotope dilution assay with GC–tandem quadrupole mass spectrometry. By correlating the sensory evaluation values of retronasal aroma, γ-hexalactone, γ-d2ecalactone, and γ-undecalactone showed a high correlation with the Wagyu beef aroma. Metabolomics data revealed a high correlation between the amounts of odorants and multiple metabolites, such as glutamine, decanoic acid, lactic acid, and phosphoric acid. These results provide useful information for assessing the aroma and quality of beef.

## 1. Introduction

Beef from Japanese Black cattle, also known as Japanese Wagyu, is one of the most renowned beef types [1]. The genetic value and meat quality of Japanese Black cattle have attracted attention worldwide [2,3,4]. Japanese Black cattle have a unique marbling derived from intramuscular fat in the muscle. Holstein cattle are a breed of dairy steers and provide lean beef that is often experimentally compared to beef from Japanese Black cattle. Japanese Black cattle and Holstein cattle are significantly different in muscle and intramuscular fat development, resulting in different marbling proportions [5]. This marbling content is an essential factor in determining the flavor, juiciness, and tenderness of meat [6,7,8]. Sensory evaluations revealed that beef from Japanese Black cattle has a higher sensory score for a rich and sweet aroma (so-called Wagyu beef aroma unique to Japanese Black beef) than beef derived from other breeds [9]. Many studies have identified odorants of this sweet flavor [10]. Matsuishi et al. [11] revealed that lactones, such as γ-nonalactone, contributed to Wagyu beef aroma using a combination of simultaneous distillation extraction and gas chromatography–mass spectrometry (GC-MS). Subsequent research on volatile odorants in Wagyu beef aroma used headspace techniques with solid-phase microextraction and simplified the aroma analysis [12,13]. In addition, the solvent-assisted flavor evaporation (SAFE) method enabled efficient collection by distillation of high-volatile odorants, such as lactones [14]. A quantitative analysis of lactones in beef has also been attempted by combining conventional solvent extraction and highly sensitive GC-tandem quadrupole mass spectrometry (MS/MS) [15]. Various studies have shown the components and concentration of odorants in Wagyu beef aroma, but data variations exist due to the different extraction methods [15]. Therefore, the compounds used as specific indicators for Wagyu beef aroma quality have not yet been identified. Gas chromatography–olfactometry (GC-O) technology combines gas chromatography analysis with sensory detection to identify odorants from a complex mixture of flavor compounds [16]. GC-O technology has already been widely used for evaluating the aroma of foods [17,18] and is effective for evaluating the characteristics of volatile components; thus, it is expected to be applied to the analysis of the aroma components of beef. Moreover, by combining the compounds in foods, it is expected to elucidate the precursors involved in generating the aroma components [19].

Our previous study performed metabolomics analysis using GC-MS to compare two different pedigrees of Japanese Black cattle with statistical analysis between metabolites and retronasal aroma [20]. Metabolomics analysis has the advantage of detecting a large number of compounds, and it can also be combined with other analytical data to expand multi-omics analysis [21]. These approaches excel in identifying critical odorants involved in food flavor from many candidates [22].

This study identified odorants that could indicate the premium quality of Wagyu beef aroma. Furthermore, metabolites that were positively correlated with the odorants were identified by collating comprehensive metabolite data obtained using metabolomics analysis. In this study, to suppress bias, such as that due to volatilization and lactone conversion on account of heating during volatile isolation and preconcentration, the aroma fraction of a ribeye steak of Japanese Black cattle was distilled using the SAFE apparatus at ultra-low temperatures. Furthermore, the distillate was analyzed by GC-O analysis.

## 2. Results and Discussion

### 2.1. Sensory Evaluation of Beef Aroma

We prepared three different types of beef to examine Wagyu beef aroma (Table 1). 

**Figure 1:**
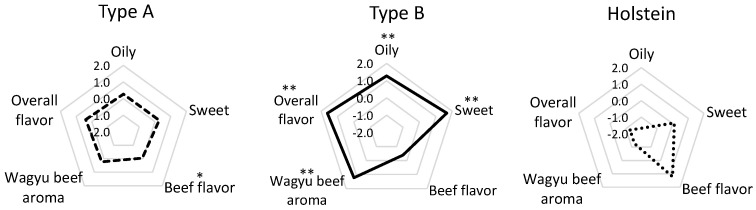
Characteristics of beef aroma by sensory evaluation of Japanese Black and Holstein cattle. The radar chart indicates the flavor intensity of sensory evaluation of grilled beef (mean, 6 cattle for each type). A significant difference was estimated using Student’s t-test between Japanese Black cattle Type A and Type B. ** Significant difference (*p* < 0.01), * significant difference (*p* < 0.05).

Type B was highly evaluated for its sweet, oily, Wagyu beef aroma (Figure 1). However, beef flavor (meaty aroma derived from lean meat) was high in Holstein cattle and Type A [23]. This result was similar to our previous sensory evaluation data obtained with boiled beef [20].

### 2.2. Establishment of Analysis Method for Beef Aroma

The beef aroma components were detected from the characteristics of the scent using GC–olfactometry. Sixty-three odor-active compounds were detected by GC–O analysis, and 39 odorants were identified by comparing their odor qualities and retention index (RI) on the DB-Wax column (Table 2). Of the 39 compounds, 8 compounds (**1**, **2**, **12**, **14**, **15**, **21**), including methional (**10**) and 4,5-epoxy-2(E)-decenal (**26**), were detected in Japanese Black cattle using GC–O technology in a study by Inagaki et al. [14].

Fifteen odorants (**5**, **7**, **8**, **9**, **10**, **11**, **12**, **16**, **17**, **21**, **22**, **26**, **29**, **30**, and **31**) had a strong aroma with flavor dilution (FD) factors greater than 4 in Japanese Black and Holstein cattle meat. According to GC-O analysis, the common aroma composition was mainly aldehyde-derived aromas and lactones. In the comparison of FD factors, 17 odorants (**1**, **2**, **4**, **5**, **6**, **8**, **16**, **19**, **20**, **21**, **23**, **27**, **29**, **31**, **32**, **36**, and **38**) were detected at higher FD values in Japanese Black cattle than in Holstein cattle. This suggested that these odorants contributed to the beef flavor of Japanese Black cattle. Of the 39 odorants, 4 odorants, hexanoic acid (**22**), maltol (**25**), 4-vinyl phenol (**33**), and decanoic acid (**34**), were detected at higher FD values in Holstein cattle (Table 2). In the comparison between cattle species, 11 odorants, octanal (**4**), 2-acetyl-1-pyrroline (**6**), butyric acid (**14**), γ-hexalactone (**16**), γ-heptalactone (**20**), γ-octalactone (**23**), γ-nonalactone (**27**), δ-decalactone (**31**), 2-aminoacetophenone (**32**), indole (**36**), and 3-methylindole (**38**), were detected at higher FD values in Type B than in Type A Japanese Black cattle and Holstein cattle. In the comparison between Japanese Black cattle, in addition to the 11 odorants (**4**, **6**, **14, 16**, **20**, **23**, **27**, **31**, **32**, **36**, and **38**) mentioned above, 4 odorants (**13**, **24**, **30**, and **39**) were detected at higher FD values in Type B than in Type A. This suggested that 11 unique odorants (**4**, **6**, **14**, **16**, **20**, **23**, **27**, **31**, **32**, **36**, and **38**) are important components of the Wagyu beef aroma. We selected γ-hexalactone (**16**), γ-heptalactone (**20**), γ-octalactone (**23**), γ-nononalactone (**27**), γ-decalactone (**29**), δ-decalactone (**31**), and vanillin (**39**) as candidates contributing to the boiled Wagyu beef aroma. Although γ-undecalactone (ND) could not be separated by GC–O, we made inferences based on its odor quality (coconut-like sweet scent) [24] and added γ-undecalactone as a candidate contributor to boiled Wagyu beef aroma. 

**Figure 2:**
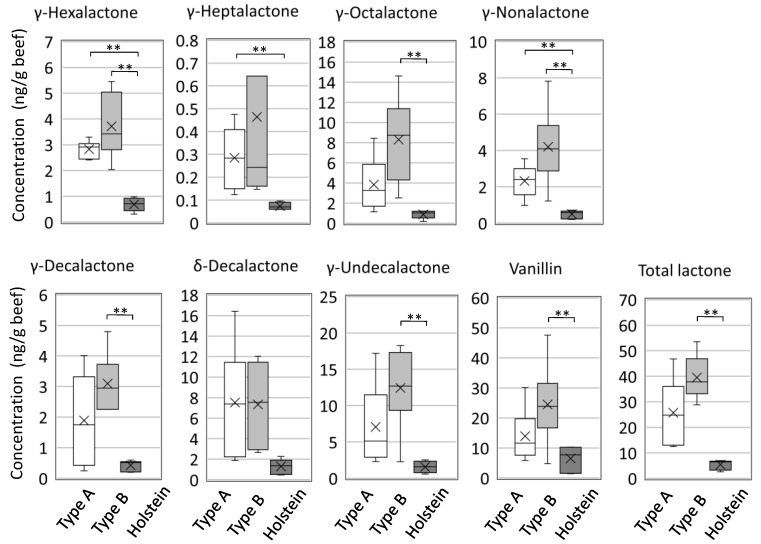
Quantification of critical odorants of the rich and sweet aromas in Japanese Black cattle meat. The quantitative values of odorants were measured using stable isotopes. The cross mark indicates the position of the mean value (6 cattle for each type). The amount of total lactone is the sum of the amounts of γ-hexalactone, γ-heptalactone, γ-octalactone, γ-nonalactone, γ-decalactone, δ-decalactone, and γ-undecalactone. A significant difference was examined using the Bonferroni test. ** Significant difference (*p* < 0.017).

Next, the eight odorants in the beef were quantified using the stable isotope dilution assay (SIDA) method with GC–tandem quadrupole mass spectrometry (GC/MS/MS). The calibration curves prepared using the stable isotope to quantify each odorant showed high quantification (*r* > 0.99, results not shown) in the GC/MS/MS apparatus. Box plots of the eight odorants are shown in Figure 2. The mean concentrations are shown in the Table 3. As expected, the eight odorants were detected at higher levels in Japanese Black cattle than in Holstein cattle. Seven odorants, excluding δ-decalactone, were detected at higher levels in Type B than those in Type A. γ-Hexalactone and γ-nonalactone showed a high significant difference (*p* < 0.005) between Japanese Black cattle and Holstein cattle. However, the eight odorants did not show a significant difference between Type A and Type B. These data are consistent with the distribution pattern of γ-lactones found in a comparison of lean meats and Japanese Black cattle in a previous report [15]. Of the eight odorants, vanillin, γ-octalactone, δ-decalactone, and γ-undecalactone were present at high levels, but these odorants have a large coefficient of variation (CV) value among Japanese Black cattle. Additionally, γ-undecalactone showed long lower whiskers on the boxplot. This variation may be the reason why γ-undecalactone was not detected by GC–O.

### 2.3. Relationship between Sensory Evaluation and Odorant

Next, the relationship between sensory evaluation and odorants was examined. As expected, the eight odorants discussed previously showed a positive correlation with Wagyu beef aroma and a negative correlation with beef flavor from both grilled and boiled beef (Table 4). γ-Hexalactone (*r* = 0.801), γ-decalactone (*r* = 0.765), γ-undecalactone (*r* = 0.765) and total lactone (*r* = 0.844) had a high positive correlation with Wagyu beef aroma from grilled beef. This correlation with odorants and flavor had a similar trend for the flavor of boiled beef. Even in boiled beef, γ-hexalactone (*r* = 0.773), γ-decalactone (*r* = 0.719), γ-undecalactone (*r* = 0.659), and total lactone (*r* = 0.795) had a high positive correlation with Wagyu beef aroma.

**Figure 3:**
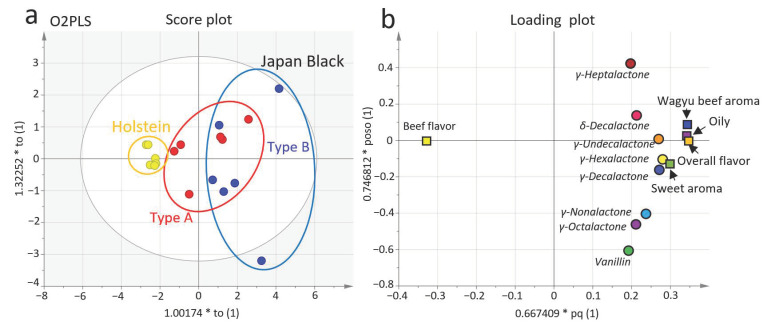
Relationship between odorants and sensory evaluation of flavor. (**a**) Two-way orthogonal partial least squares (O2PLS) score plot visualizing the relationship among Japanese Black cattle Type A, Type B, and Holstein cattle using odorant data for X variables, and the results of the sensory evaluation of grilled beef flavor for Y variables (each type of cattle, *n* = 6; scaling, UV). (**b**) The loading plot exhibits the relationship between odorants and flavor from the sensory evaluation. The O2PLS model was used for analysis with fitting parameters (components, (1 + 2); R2X (1) = 0.604; R2X (2) = 0.167).

For further investigation, the data were analyzed using multiple variables and two-way orthogonal partial least squares (O2PLS) (Figure 3). The loading plot distribution demonstrated the strength of the relationship between the odorant and sensory evaluation. γ-Hexalactone, γ-decalactone, and γ-undecalactone were closely clustered with the quality of sweet aroma and contributed to Wagyu beef aroma, oily, and the overall flavor.

However, vanillin and γ-heptalactone were not related to Wagyu beef aroma. This result suggested that γ-hexalactone, γ-decalactone, and γ-undecalactone were key odorants with a particularly excellent Wagyu beef aroma from Type B Japanese Black cattle. Thus, γ-hexalactone, which has a low CV value in the analysis (Table 3), could serve as a potential marker to estimate the degree of Wagyu beef aroma.

### 2.4. Identification of Metabolites Associated with Rich and Sweet Aromas

**Figure 4:**
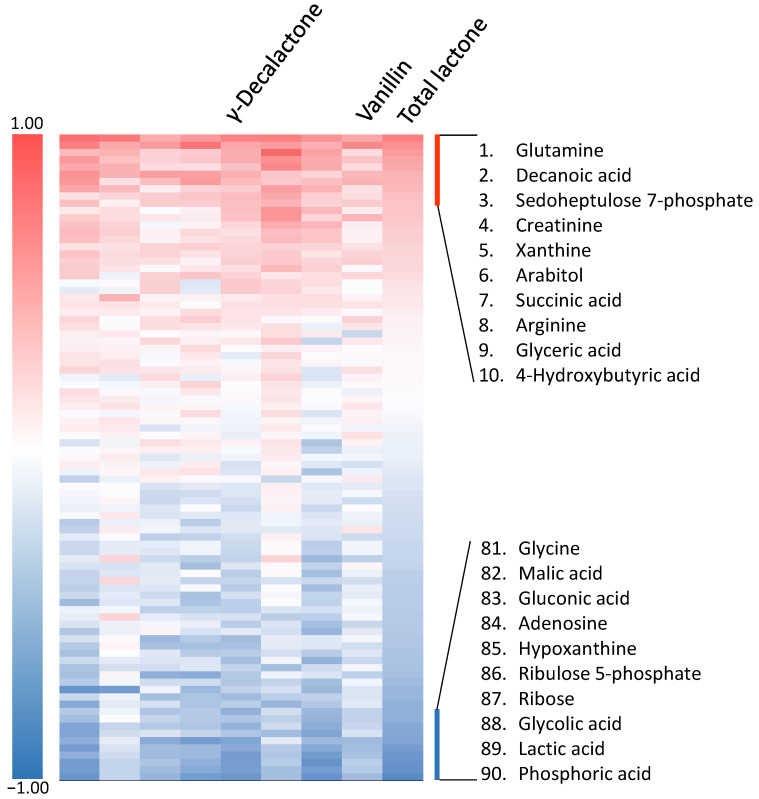
Heatmap summarizing the relationship between odorants and metabolites in beef. Pearson’s correlation coefficient between the quantitative value of odorants and metabolites by GC/MS. Red indicates a strong positive correlation, and blue indicates a strong negative correlation. The ninety metabolites are ranked from positive to negative based on the correlation coefficient of the total amount of lactone.

The composition of water-soluble nutrition in beef is important for the Maillard reaction related to aroma characteristics [25]. The identification of Wagyu beef aroma precursors to provide a new added value is an interesting subject in the livestock market. Previously, metabolomic analysis was performed, which identified 90 metabolites comprising amino acids, organic acids, and carbohydrates in Japanese Black cattle (Types A and B) and Holstein cattle [20]. These samples were frozen-stocked and showed that the signal intensities of many organic acids and amino acids were 50% lower in Type B than in Type A, and significant differences were observed in 31 metabolites (Type A vs. Type B) and 19 metabolites (Type B vs. Holstein) [20]. The correlation coefficient between the amounts of the eight odorants and the metabolites was calculated. A heatmap ranking indicated the order from positive to negative, based on the correlation coefficient of total lactone. The top 10 metabolites with the highest positive or negative correlation are listed on the heatmap in Figure 4. Of the eight odorants, γ-hexalactone showed a relatively high similarity to the pattern of total lactone. This γ-hexalactone also showed a high correlation with glutamine, decanoic acid, phosphoric acid, glycolic acid, lactic acid, ribulose 5-phosphate, hypoxanthine, and adenosine (Figure S1). Among the positively correlated metabolites, decanoic acid is abundant in intramuscular fat and has been proposed as a molecular indicator to estimate the degree of intramuscular fat [20]. Consistent with various studies [4,14,15], analysis of decanoic acid also indirectly showed that intramuscular fat is related to rich and sweet aroma. Indeed, branched-chain amino acids, which are indicators of lean meat, showed a conversely low correlation with lactones (leucine: *r* = −0.095, isoleucine: *r* = 0.047, methionine: *r* = −0.267). Other positive metabolites, glutamine, sedoheptulose 7-phosphate, creatinine, succinic acid, and arginine, have been reported to increase gradually from early postmortem aging [26]. Glutamine is known to provide an umami taste, and its concentration is closely related to the favorite aroma of beef [27,28,29]. High concentrations of creatinine delay lactic acid accumulation, which is known as the final metabolite of glycolysis in postmortem aging [28,30]. These positive metabolites may contribute to the synthesis of lactones and the differences in aroma characteristics [20,31]. However, phosphoric acid, lactic acid, glycolic acid, and hypoxanthine showed a highly negative correlation with γ-hexalactone (Figure 4). Phosphoric acid and hypoxanthine are metabolites derived from inosine monophosphate (IMP); IMP is also a nucleotide related to the umami taste and is degraded into hypoxanthine during postmortem aging [32]. These results suggested that proper meat storage and the aging period affect the Wagyu beef aroma. 

## 3. Materials and Methods

### 3.1. Sample Collection

Japanese Black cattle have three significant pedigrees (*Tajima, Kedaka, Fujiyoshi*), depending on the production area [1]. In this study, we selected two representative production areas of Japanese Black cattle, Type A and Type B [33]. Holstein cattle are dairy steers and were used due to their low marbling as a control sample of lean meat for Japanese Black cattle (Table 1). All three groups of cattle were steers and grain-fed, and slaughtered in the same commercial slaughterhouse. Type A is a common Japanese Black cattle pedigree and exhibits excellent bodyweight growth. Type B is a closed breeding breed in Hyogo prefecture [34] and is a grade of a Kobe beef [35] that is highly traded with excellent meat quality. 

Blocks of longissimus muscle of Japanese Black cattle and Holstein cattle were vacuum-packed at the Meat Processing Center (Himeji, Japan) and stored at 4 °C for 20 days after slaughter. The blocks were sliced into 1-cm-thick steaks from the front and were individually packaged for sensory evaluation, quantitative analysis of odorants, and metabolomics analysis.

These samples were frozen-stocked at −30 °C for 1 week until all samples were collected. The samples were subjected to experimentation within 2 weeks to prevent deterioration due to long-term freezing. After slow thawing of each sample at 4 °C overnight, the ribeye portion of loin (200–250 g) was analyzed (total postmortem time, 21 days).

### 3.2. Sensory Evaluation

We outsourced the analysis of the beef aroma characteristics of Japanese Black and Holstein cattle to the Japan Meat Science and Technology Institute (Tokyo, Japan). Three professional analytical panelists who routinely perform sensory evaluation tests, particularly on meat, evaluated the retronasal aroma of Japanese Black cattle Type A and Type B and Holstein cattle (six cattle of each type). A barbecue-cut sample (40 mm × 50 mm, thickness: 10 mm) was placed on a vat and exposed to air overnight in a refrigerator at 4 °C. The sample was cooked as a grilled steak by heating at 230 °C for 60 s and then cooking the backside for 90 s with an electric griddle (Zojirushi, Tokyo, Japan). In compliance with the sensory evaluation guidelines of meat (National Livestock Breeding Center, Tokyo, Japan), cooked beef was provided as blind samples one at a time in the sensory evaluation room (2–3 samples per day). Sensory evaluation was performed on five items: oily, sweet, beef flavor (roasted lean meat), Wagyu beef aroma (favorable rich and sweet coconut-like aroma) [11,36], and overall flavor, with the evaluation scale ranging from −3.00 to +3.00. The trained panelists used a nose clip to judge the flavor; the clip was removed after chewing. The evaluation scale of the separately prepared reference beef (corresponding to Japanese Black cattle Type A) was set to 0.00.

### 3.3. Concentration of Odorants from Boiled Beef

A total of 50 g of ribeye from steak was boiled in 500 mL of distilled water for 30 s. The sample was cooled with ice, ground with a mixer, added to 500 mL of dichloromethane, and extracted for 16 h at 25 °C with stirring. In the solvent extraction of Japanese Black cattle, the dichloromethane layer contained many nonvolatile constituents, such as fats and oils. To remove them from the sample, a SAFE apparatus was used that distilled the dichloromethane extract at ultra-low temperature to minimize the volatilization of aroma components. The distillation temperature was kept at cryogenic levels with liquid nitrogen to minimize the volatilization of aroma components. [37]. After drying with anhydrous sodium sulfate, the sample was concentrated to a final volume of 200 µL using a Kuderna-Danish Evaporative Concentrator. 

### 3.4. GC–O Analysis

Prior to the GC–O analysis, five trained sensory panelists performed a flavor-profile analysis of the odorants from boiled beef [38]. The aroma characteristics of Wagyu beef aroma were selected by comparing Japanese Black cattle Type A and Type B and Holstein cattle. The typical characteristics of expressing Wagyu beef aroma were “sweet aroma”, “milk-like aroma”, and “rich flavor”. Before GC–O analysis, the concentrated sample was organoleptically checked to determine if it retained the original aroma characteristics. The GC–O analysis was performed by CharmAnalysis (DATU, Geneva, NY, USA) using an Agilent 6890 gas chromatograph (Agilent Technologies, Santa Clara, CA, USA) equipped with a DB-WAX capillary column (length 15 m; inner diameter 0.32 mm; film thickness 0.25 mm; Agilent Technologies) [39]. The humidified airflow to the sniff port was set to 30 mL/min at 60 °C. The oven temperature was programmed to rise from 40 °C to 230 °C at a rate of 6 °C/min and was held at 230 °C for 20 min. The odor extract was diluted stepwise (4-fold) with dichloromethane. The FD factor was determined under the supervision of two experts in the field of beer hop aromas [38,39,40]. Identification of compounds was performed by comparing their odor qualities, RIs, and mass spectra with those of authentic compounds on a DB-WAX column.

### 3.5. Quantification of Odorants

The concentration of odorous components was measured by stable isotope dilution assay (SIDA) using a liquid extraction method in which stable isotopes were added to dichloromethane as internal standards. Stable isotopes of 2-acetylpyroline, vanillin, γ-nonalactone, γ-octalactone, γ-decalactone, γ-undecalactone, γ-hexalactone, γ-heptalactone, and δ-decalactone were purchased from AromaLAB (Martinsried, Germany). Quantitation was performed using a GC–tandem quadrupole mass spectrometer (Agilent 7000C TripleQuad GC/MS system, Agilent Technologies) equipped with a DB-WAX capillary column (length, 30 m; inner diameter, 0.25 mm; film thickness, 0.25 mm; Agilent Technologies). The oven temperature was programmed to hold at 35 °C for 5 min and was then increased from 35 °C to 217 °C at a rate of 4 °C/min. The system was operated in multiple reaction monitoring (MRM) mode. Two microliters of the concentrate were injected into the instrument with the inlet temperature set at 250 °C in the spitless mode.

### 3.6. Metabolomics Analysis

Previous metabolomics data were used in this study. Briefly, a water-soluble fraction was extracted from the same beef sample (6 cattle for each type) and subjected to GC/MS analysis (GCMS-QP2010 Ultra, Shimadzu Corporation, Kyoto, Japan) through a conventional method [20].

### 3.7. Statistical Analysis

Statistical significance was determined using a *p-*value (Student’s *t*-test and Bonferroni test). The correlation coefficient between sensory evaluation and odor was calculated via a single-phase analysis. Multivariate data analysis of O2PLS was performed using the SIMCA14 software (Inforcom, Tokyo, Japan).

## 4. Conclusions

This study identified more than 39 odorants using GC–O analysis and succeeded in quantifying 8 odorants contributing to Wagyu beef aroma. These results indicated that γ-hexalactone, γ-decalactone, and γ-undecalactone contributed significantly to Wagyu beef aroma. Thus, γ-hexalactone is proposed as a marker of Wagyu beef aroma. Additionally, several critical metabolites related to Wagyu beef aroma were identified by integrating these data with those of our previous metabolomics study. Glutamine, decanoic acid, phosphoric acid, lactic acid, creatinine, and hypoxanthine were considered important indicators of the quality of Wagyu meat.

## Figures and Tables

**Table 1:** Details of beef samples used for testing.

Group	Pedigree	Number of Cattle	Production Area	Slaughtered Age (month)	Carcass Weight (kg)
Type A	Kedaka, Fujiyoshi, Yasufuku	6	Kagoshima prefecture	29.5 ± 0.9	483.4 ± 40.1
Type B	Tajima	6	Hyogo prefecture	32.3 ± 1.3	406.6 ± 18.5
Holstein	Holstein	6	Tochigi Prefecture	21.3 ± 2.4	451.6 ± 47.9

**Table 2:** Identification of 39 odorants in Japanese Black cattle meat.

No.	^a^ RI	Conpound	^c^ Odor Quality	^b^ FD Factor (Log_4_)		
				Japanese Black Cattle		Holstein
				Type A	Type B	
1	983	2,3-Butanedione	Buttery	4	4	2
2	1105	Hexanal	Fresh leaves	5	5	3
3	1123	3-Methyl-2-butene-1-thiol	Burnt	-	-	<1
4	1291	Octanal	Green fresh	2	4	2
5	1299	2-Methyl-3-furanthiol	Nuts	7	7	5
6	1331	2-Acetyl-1-pyrroline	Grain	1	4	1
7	1371	1,5-Octadien-3-one	Green, Metallic	5	4	5
8	1423	Nonanal	Oil oxidation	7	7	5
9	1438	(E)-2-octenal	Grassy-smelling	5	5	5
10	1441	Methional	Stewed potatoes	7	6	7
11	1501	Decanal	Green fresh	7	7	7
12	1530	(E)-2-Nonenal	Oil oxidation	7	7	7
13	1577	(2E,6Z)-Nona-2,6-dienal	Cucumber	2	4	4
14	1623	Butyric acid	Cheese odor	1	4	2
15	1669	Isovaleric acid	Cheese odor	2	-	2
16	1684	γ-Hexalactone	Sweet milk	6	7	6
17	1690	(2E,4E)-2,4-Nonadienal	Oil oxidation	7	7	7
18	1731	2-Acetyl-1-thiazoline	Grain	3	3	3
19	1757	2-Undecenal	Oil oxidation	4	2	2
20	1787	γ-Heptalactone	Sweet milk	1	4	-
21	1800	(E,E)-2,4-Decadienal	Oil oxidation	7	7	5
22	1842	Hexanoic acid	Dust cloth	6	5	7
23	1890	γ-Octalactone	Lactone, Sweet Scent	4	7	3
24	1928	β-Ionone	Violet	<1	2	2
25	1941	Maltol	Sweet yogurt	3	3	6
26	1989	4,5-Epoxy-2(E)-decenal	Metal	7	7	7
27	2004	γ-Nonalactone	Lactone, Sweet Scent	4	5	2
28	2021	Franeol	Sweet yogurt	5	3	5
29	2099	γ-Decalactone	Lactone, Sweet Scent	7	7	6
30	2171	4-Vinyl guaiacol	Smoky	6	7	7
31	2185	δ-Decalactone	Lactone, Sweet Scent	5	7	5
32	2188	2-Aminoacetopheone	Grape	2	3	2
33	2256	4-Vinyl phenol	Smoky	<1	-	3
34	2288	Decanoic acid	Dust cloth	4	4	6
35	2361	9-Decenoic acid	Dust cloth	<1	-	<1
36	2368	Indole	Indole	3	4	3
37	2445	3-Methoxyphenol	Vanilla	1	1	1
38	2459	3-Methylindole	Indole	<1	1	<1
39	2537	Vanillin	Chocolate, vanilla	5	6	6

^a^ RI is the retention index of the DB-WAX column used in the gas chromatography–mass spectrometry (GC–MS). ^b^ Flavor dilution (FD) factors determined under the supervision of scent experts. ^c^ Fragrance detected at the sniffing port during gas chromatography–olfactometry (GC-O).

**Table 3:** The table shows the mean quantification values (ng/50 g beef) and the standard deviations. Numbers in parentheses indicate the coefficient of variation (CV) values. The quantitative analysis was performed on the same samples for which sensory evaluation was performed in Figure 1.

	γ-Hexalactone	γ-Heptalactone	γ-Octalactone	γ-Nonalactone
**Japanese Black**				
Type A	141.3 ±16.9 (0.12)	14.2 ± 6.8 (0.48)	191.5 ± 131.3 (0.69)	115.8±44.2 (0.38)
Type B	185.9 ± 63.3 (0.34)	23.2 ± 30.3 (1.30)	414.9 ± 215.7 (0.52)	210.0 ± 106.9 (0.51)
Holstein	34.3 ± 13.0 (0.38)	3.7 ± 0.8 (0.22)	43.8 ± 20.3 (0.46)	24.9±10.9(0.44)
**Japanese Black**				
Type A	94.3 ± 73.2 (0.78)	376.6 ± 267.9 (0.71)	353.5 ± 278.4 (0.79)	696.6 ± 436.3 (0.63)
Type B	154.8 ± 47.2 (0.30)	367.1 ± 197.5 (0.54)	621.7 ± 281.7 (0.45)	1224.7 ± 686.0 (0.56)
Holstein	21.3 ± 8.8 (0.41)	64.8 ± 36.7 (0.57)	78.2 ± 38.7 (0.49)	324.1 ± 201.1 (0.62)

**Table 4:** The correlation coefficient between sensory evaluation and odorants. The table shows the Pearson’s correlation coefficient between the flavor intensity of the sensory evaluation and the quantitative value of odorants.

**Grilled Beef Flavor**	**γ-Hexalactone**	**γ-Heptalactone**	**γ-Octalactone**	**γ-Nonalactone**	**γ-Decalactone**	**δ-Decalactone**	**γ-Undecalactone**	**Vanillin**	**Total Lactone**
Oily	0.818	0.467	0.637	0.653	0.730	0.537	0.712	0.467	0.798
Sweet	0.657	0.422	0.624	0.657	0.674	0.347	0.610	0.520	0.684
Beef flavor	−0.882	−0.489	−0.606	−0.695	−0.732	−0.624	−0.764	−0.550	−0.846
Wagyu beef aroma	0.801	0.508	0.618	0.662	0.765	0.636	0.765	0.429	0.844
Overall flavor	0.807	0.464	0.648	0.695	0.763	0.560	0.727	0.492	0.820
**Boiled Beef Flavor**	**γ-Hexalactone**	**γ-Heptalactone**	**γ-Octalactone**	**γ-Nonalactone**	**γ-Decalactone**	**δ-Decalactone**	**γ-Undecalactone**	**Vanillin**	**Total Lactone**
Oily	0.786	0.470	0.604	0.642	0.729	0.541	0.697	0.437	0.782
Sweet	0.765	0.474	0.653	0.709	0.746	0.446	0.701	0.506	0.776
Beef flavor	−0.837	−0.400	−0.637	−0.699	−0.662	−0.487	−0.692	−0.497	−0.777
Wagyu beef aroma	0.773	0.408	0.661	0.648	0.719	0.601	0.659	0.462	0.795
Overall flavor	0.771	0.446	0.607	0.627	0.739	0.604	0.688	0.445	0.794

## Data Availability

The data presented in this study are available in article and supplementary material.

## Supplementary Materials

The following are available online at https://www.mdpi.com/2218-1989/11/1/56/s1, Figure S1: Relationship between γ-hexalactone and metabolites in the beef of Japanese Black cattle Type A and Type B and Holstein cattle. The graph shows a scatter plot of metabolites that correlate with γ-hexalactone (each type of cattle, n = 6).

## References

[1] Motoyama M., Sasaki K., Watanabe A. (2016). Wagyu and the factors contributing to its beef quality: A Japanese industry overview. Meat Sci..

[2] Zhao G., Zhang T., Liu Y., Wang Z., Xu L., Zhu B., Gao X., Zhang L., Gao H., Liu G.E. (2020). Genome-Wide Assessment of Runs of Homozygosity in Chinese Wagyu Beef Cattle. Animals.

[3] Scraggs E., Zanella R., Wojtowicz A., Taylor J.F., Gaskins C.T., Reeves J.J., de Avila J.M., Neibergs H.L. (2014). Estimation of inbreeding and effective population size of full-blood Wagyu cattle registered with the American Wagyu Cattle Association. J. Anim. Breed. Genet..

[4] Frank D., Ball A., Hughes J., Krishnamurthy R., Piyasiri U., Stark J., Watkins P., Warner R. (2016). Sensory and Flavor Chemistry Characteristics of Australian Beef: Influence of Intramuscular Fat, Feed, and Breed. J. Agric. Food Chem..

[5] Gotoh T., Nishimura T., Kuchida K., Mannen H. (2018). The Japanese Wagyu beef industry: Current situation and future prospects-A review. Asian Australas. J. Anim. Sci..

[6] Watanabe G., Motoyama M., Orita K., Takita K., Aonuma T., Nakajima I., Tajima A., Abe A., Sasaki K. (2019). Assessment of the dynamics of sensory perception of Wagyu beef strip loin prepared with different cooking methods and fattening periods using the temporal dominance of sensations. Food Sci. Nutr..

[7] Corbin C.H., O’Quinn T.G., Garmyn A.J., Legako J.F., Hunt M.R., Dinh T.T.N., Rathmann R.J., Brooks J.C., Miller M.F. (2015). Sensory evaluation of tender beef strip loin steaks of varying marbling levels and quality treatments. Meat Sci..

[8] Shahrai N.N., Babji A.S., Maskat M.Y., Razali A.F., Yusop S.M. (2020). Effects of marbling on physical and sensory characteristics of ribeye steaks from four different cattle breeds. Asian Australas. J. Anim. Sci..

[9] Matsuishi M., Fujimori M., Okitani A. (2001). Wagyu Beef Aroma in Wagyu (Japanese Black Cattle) Beef Preferred by the Japanese over Imported Beef. Anim. Sci. J..

[10] Maruri J.L., Larick D.K. (1992). Volatile Concentration and Flavor of Beef as Influenced by Diet. J. Food Sci..

[11] Matsuishi M., Kume J., Itou Y., Takahashi M., Arai M., Nagatomi H., Watanabe K., Hayase F., Okitani A. (2004). Aroma Components of Wagyu Beef and Imported Beef. Nihon Chikusan Gakkaiho.

[12] Watanabe A., Ueda Y., Higuchi M., Shiba N. (2008). Analysis of Volatile Compounds in Beef Fat by Dynamic-Headspace Solid-Phase Microextraction Combined with Gas Chromatography–Mass Spectrometry. J. Food Sci..

[13] Watanabe A., Kamada G., Imanari M., Shiba N., Yonai M., Muramoto T. (2015). Effect of aging on volatile compounds in cooked beef. Meat Sci..

[14] Inagaki S., Amano Y., Kumazawa K. (2017). Identification and Characterization of Volatile Components Causing the Characteristic Flavor of Wagyu Beef (Japanese Black Cattle). J. Agric. Food Chem..

[15] Yoshinaga K., Tago A., Yoshinaga-Kiriake A., Gotoh N. (2021). Characterization of lactones in Wagyu (Japanese beef) and imported beef by combining solvent extraction and gas chromatography–mass spectrometry. LWT.

[16] Delahunty C.M., Eyres G., Dufour J.P. (2006). Gas chromatography-olfactometry. J. Sep. Sci..

[17] d’Acampora Zellner B., Dugo P., Dugo G., Mondello L. (2008). Gas chromatography–olfactometry in food flavour analysis. J. Chromatogr. A.

[18] Brattoli M., Cisternino E., Dambruoso P.R., de Gennaro G., Giungato P., Mazzone A., Palmisani J., Tutino M. (2013). Gas chromatography analysis with olfactometric detection (GC-O) as a useful methodology for chemical characterization of odorous compounds. Sensors.

[19] Diez-Simon C., Mumm R., Hall R.D. (2019). Mass spectrometry-based metabolomics of volatiles as a new tool for understanding aroma and flavour chemistry in processed food products. Metab. Off. J. Metab. Soc..

[20] Ueda S., Iwamoto E., Kato Y., Shinohara M., Shirai Y., Yamanoue M. (2019). Comparative metabolomics of Japanese Black cattle beef and other meats using gas chromatography-mass spectrometry. Biosci. Biotechnol. Biochem..

[21] Goldansaz S.A., Guo A.C., Sajed T., Steele M.A., Plastow G.S., Wishart D.S. (2017). Livestock metabolomics and the livestock metabolome: A systematic review. PloS ONE.

[22] Muroya S., Ueda S., Komatsu T., Miyakawa T., Ertbjerg P. (2020). MEATabolomics: Muscle and Meat Metabolomics in Domestic Animals. Metabolites.

[23] Kerth C. (2016). Determination of volatile aroma compounds in beef using differences in steak thickness and cook surface temperature. Meat Sci..

[24] Pérez-Olivero S.J., Pérez-Pont M.L., Conde J.E., Pérez-Trujillo J.P. (2014). Determination of lactones in wines by headspace solid-phase microextraction and gas chromatography coupled with mass spectrometry. J. Anal. Methods Chem..

[25] Khan M.I., Jo C., Tariq M.R. (2015). Meat flavor precursors and factors influencing flavor precursors—A systematic review. Meat Sci..

[26] Muroya S., Oe M., Ojima K., Watanabe A. (2019). Metabolomic approach to key metabolites characterizing postmortem aged loin muscle of Japanese Black (Wagyu) cattle. Asian Australas. J. Anim. Sci..

[27] Kim Y.H.B., Kemp R., Samuelsson L.M. (2016). Effects of dry-aging on meat quality attributes and metabolite profiles of beef loins. Meat Sci..

[28] Antonelo D.S., Cônsolo N.R.B., Gómez J.F.M., Beline M., Goulart R.S., Corte R.R.P.S., Colnago L.A., Schilling M.W., Gerrard D.E., Silva S.L. (2020). Metabolite profile and consumer sensory acceptability of meat from lean Nellore and Angus × Nellore crossbreed cattle fed soybean oil. Food Res. Int..

[29] Dang Y., Gao X., Ma F., Wu X. (2015). Comparison of umami taste peptides in water-soluble extractions of Jinhua and Parma hams. LWT Food Sci. Technol..

[30] Nissen P.M., Young J.F. (2006). Creatine Monohydrate and Glucose Supplementation to Slow- and Fast-Growing Chickens Changes the Postmortem pH in Pectoralis Major. Poult. Sci..

[31] Yamada T., Kawakami S.I., Nakanishi N. (2009). Expression of adipogenic transcription factors in adipose tissue of fattening Wagyu and Holstein steers. Meat Sci..

[32] Ichimura S., Nakamura Y., Yoshida Y., Hattori A. (2017). Hypoxanthine enhances the cured meat taste. Anim. Sci. J. Nihon Chikusan Gakkaiho.

[33] Oyama K. (2011). Genetic variability of Wagyu cattle estimated by statistical approaches. Anim. Sci. J. Nihon Chikusan Gakkaiho.

[34] Oka A., Maruo Y., Miki T., Yamasaki T., Saito T. (1998). Influence of vitamin A on the quality of beef from the Tajima strain of Japanese Black cattle. Meat Sci..

[35] Kobe Beef Marketing & Distribution Promotion Association (2020). Kobe Beef. http://www.kobe-niku.jp/top.html.

[36] Iida F., Miyazaki Y., Tsuyuki R., Kato K., Egusa A., Ogoshi H., Nishimura T. (2016). Changes in taste compounds, breaking properties, and sensory attributes during dry aging of beef from Japanese black cattle. Meat Sci..

[37] Engel W., Bahr W., Schieberle P. (1999). Solvent assisted flavour evaporation–A new and versatile technique for the careful and direct isolation of aroma compounds from complex food matrices. Eur. Food Res. Technol..

[38] Kishimoto T., Wanikawa A., Kono K., Shibata K. (2006). Comparison of the Odor-Active Compounds in Unhopped Beer and Beers Hopped with Different Hop Varieties. J. Agric. Food Chem..

[39] Kishimoto T., Noba S., Yako N., Kobayashi M., Watanabe T. (2018). Simulation of Pilsner-type beer aroma using 76 odor-active compounds. J. Biosci. Bioeng..

[40] Kishimoto T., Wanikawa A., Kagami N., Kawatsura K. (2005). Analysis of hop-derived terpenoids in beer and evaluation of their behavior using the stir bar-sorptive extraction method with GC-MS. J. Agric. Food Chem..

