# Supplementary material for: Exploring the Characteristic Aroma of Beef from Japanese Black Cattle (Japanese Wagyu) via Sensory Evaluation and Gas Chromatography-Olfactometry
Source: Metabolites. 2021 Jan 15;11(1):56. doi: 10.3390/metabo11010056 (PMC7830604; doi:10.3390/metabo11010056)
Supplement: Supplementary file 1 [file metabolites-11-00056-s001.pdf]

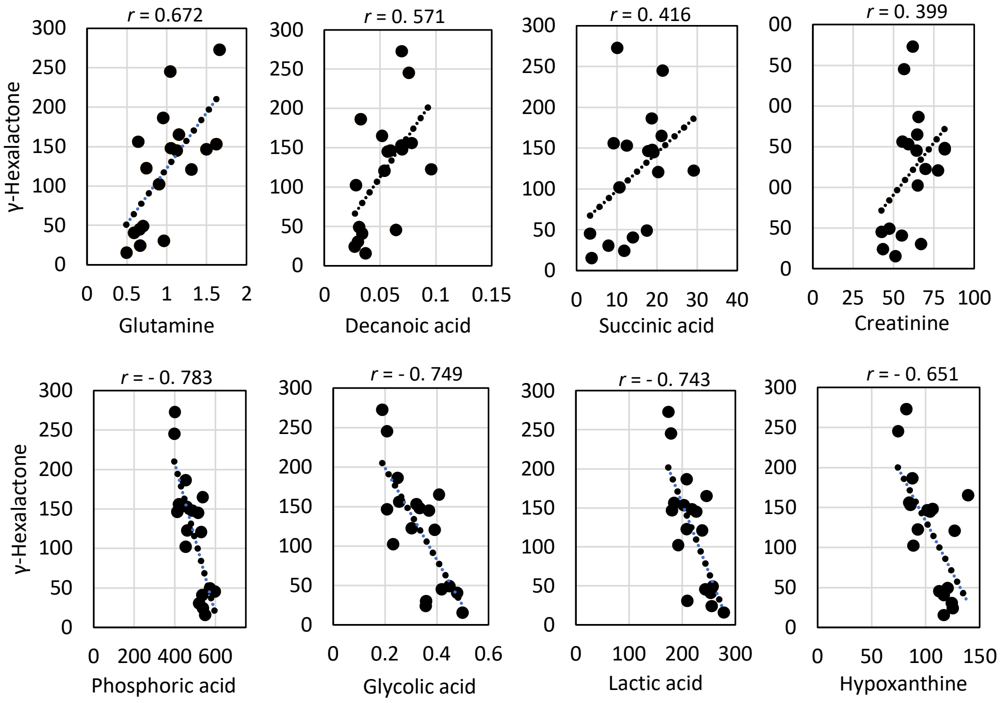

Figure S1: Relationship between γ-hexalactone and metabolites in the beef of Japanese Black cattle Type A and Type B and Holstein cattle. The graph shows a scatter plot of metabolites that correlate with γ-hexalactone (each type of cattle, n = 6).
